# Characterization and phylogenetic analysis of the complete mitochondrial genome of *Notostomum cyclostomum*

**DOI:** 10.1080/23802359.2022.2116958

**Published:** 2022-09-08

**Authors:** Jun Young Chae, Jin Ho Kim, Tae-Wook Kang, Hyung-Ho Lee, Moo-Sang Kim

**Affiliations:** a Department of Bioinformatics, MOAGEN, Daejeon, Republic of Korea; b Department of Biotechnology, Pukyong National University, Busan, Republic of Korea

**Keywords:** Mitochondrial genome, *Notostomum cyclostomum*

## Abstract

*Notostomum cyclostomum* is a parasite that lays eggs on the snow crab shells and causes various diseases by parasitizing fish. Although there have been some studies on the life history of this parasite and the associated fish diseases, little is known about the molecular biology of this parasite. Thus, here we report the mitochondrial genome of *N. cyclostomum*, which is 16,972 bp long and contains 13 mitochondrial protein-coding genes (PCGs), 22 transfer RNA (tRNA) genes, two ribosomal RNA (rRNA) genes, and a putative control region with a 92% AT-rich sequences between *tRNA-R* and *tRNA-H*. Phylogenetic analysis using 13 PCGs confirmed that *N. cyclostoum* belongs to the family *Pisciodlidae*. This is the first study revealing the complete mitochondrial genome sequence of *N. cyclostomum*.

*Notostomum cyclostomum* (Johansson 1898) is a parasite belonging to *Piscicolidae* and inhabits the East Sea of Russia (Epshtein 1996) and the northeastern Pacific Ocean of British Colombia Canada, and Alaska USA (Sloan et al. 1984; Khan and Paul 1995). *Notostomum cyclostomum* lays eggs on crustacean carapace like snow crab (*Chionoecetes opilio*) and hatches there before reaching fish. After hatching and reaching to the juvenile stage, it seeks fish for parasitizing on blood. *Notostomum cyclostomum* doesn’t affect snow crab, but as a vector it mediates infection of *Cryptobia* sp., one of hemaflagellate fish parasites (Meyers and Burton 2009). Although having a unique life cycle, little is known about genetic and molecular biology of *N. cyclostomum*. The mitogenome of *Piscicolidae* is still uncovered and only that of *Zeylanicobdella arugamensis* has been reported (Wang et al. 2018). In this study, we identified the complete mitochondrial DNA (mtDNA) sequence of *N. cyclostomum*.

The *N. cyclostomum* was obtained from the carapace of snow crab (*Chionoecetes opilio*) purchased at a fish market in Yeongdeok-gun (36°97′N, 130°10′E), South Korea, and deposited at the Pukyong National University (Voucher no. PKNU_2021_001: Jun Young Chae, jychae@pukyong.ac.kr). Genomic DNA (gDNA) was extracted from whole body of *N. cyclostomum* using Bead™ Genomic DNA Prep Kit for Animal Tissue (Biofact, Republic of Korea) according to the manufacturer recommendations. PCR was performed on *cox1* gene using the invertebrate universal primer set (Folmer et al. 1994), LCO1490 (5′-GGTCAACAAATCATAAAGATATTGG-3′), HCO2198 (5′-TAAACTT-CAGGGTGACCAAAAAATCA-3′). PCR reaction conditions were as follows: Pre-denaturation at 95 °C for 2 min, 30 cycles of 20 sec at 95 °C, 20 sec at 40 °C, and 45 sec at 72 °C and a final extension for 5 min at 72 °C. The *cox1* sequence was used to identify the species using the Basic Local Alignment Search Tool (BLAST) of the National Center for Biotechnology Information (NCBI). An NCBI BlastN (Johnson et al. 2008) search of *cox1* sequence showed 99.25% identity to *cox1* sequence (DQ414327.1) of the *N. cyclostomum*, confirming that the parasite used in this study is *N. cyclostomum*.

The genomic library for next-generation sequencing was prepared with the extracted gDNA (1 µg) using MGIEasy DNA library prep kit (MGI, China). Sequencing was conducted using MGISEQ-2000 (MGI, China) with 150 bp paired-end reads. Raw data were deposited in Sequence Read Archive (SRA) database (SRR19136323). The raw data were trimmed with Trimmomatic *ver.* 0.39 (Bolger et al. 2014), and a contig sequence was produced by default option in the *de novo* assembler of the CLC Genomics Workbench *ver.* 20.04 (QIAGEN). The circular form of mitogenome was confirmed by using ‘Map to Reference’ tool of Geneious software *ver.* 2021.2.2 (https://www.geneious.com) from mapping the filtered data into the contig sequence, and finally showed a 16,972 bp long complete mtDNA sequence of *N. cyclostomum*.

The complete mtDNA sequence was used to obtain basic annotation information using the MITOS WebServer (Bernt, Donath, et al. 2013), and the detailed annotation was manually corrected using SnapGene software (*ver.* 5.3.2, GSL Biotech LLC, snapgene.com; Wang et al. 2018). The complete mtDNA genome constituted of 13 protein-coding genes (PCGs), 22 tRNA genes, two rRNA genes, and putative control AT-rich region. All PCGs were encoded on the positive strand, and started with an ATG codon. The rest 12 PCGs, except for *nad4l* having standard TAA codon, were stopped with the truncated codons T––. The genome showed a total of 22 tRNAs including two *tRNA-L* and two *tRNA-S* genes. Predicted secondary structure of 14 tRNAs among these tRNAs showed the standard cloverleaf structure, however *tRNA-E* was with missing T-arm, and six tRNAs (*tRNA-F*, *tRNA-G*, *tRNA-H*, *tRNA-P*, *tRNA-V*, and *tRNA-Y*) and one tRNAs (*tRNA-S1*) were with missing loop structure in T-arm and D-arm, respectively. Small rRNA with a 739 bp length was located in between *tRNA-M* and *tRNA-V* and 1137 bp long large rRNA was observed in between *tRNA-V* and *tRNA-L1*. The putative control region was 2602 bp long, located in between *tRNA-R* and *tRNA-H* and possessed AT-rich region with 92% composition. This high AT composition was also observed in *Zeylanicobdella arugamensis*, the closest species among the reported mtDNA sequences (Wang et al. 2018).

The phylogenetic tree was performed within the scope of class Clitellata with 15 available mitogenomes including *Lumbricus terrestris* (U24570) and *Aporrectodea rosea* (NC_047733) as out-groups, using MrBayes *ver.* 3.2.6 (Huelsenbeck and Ronquist 2001). The nucleotide sequences of PCGs from other related species were aligned and analyzed using GTR substitution model and 1,100,000 chain length (Figure 1). The results indicate that *N. cyclostomum* is phylogenetically close to *Z. arugamensis* (NC_035308) belonging to the family Pisciolidae, while in groups refer to the two explicitly partitioned lineages into family Pisciolidae and Hirudinidae in the order Hirudinida (Figure 1).

**Figure 1.**
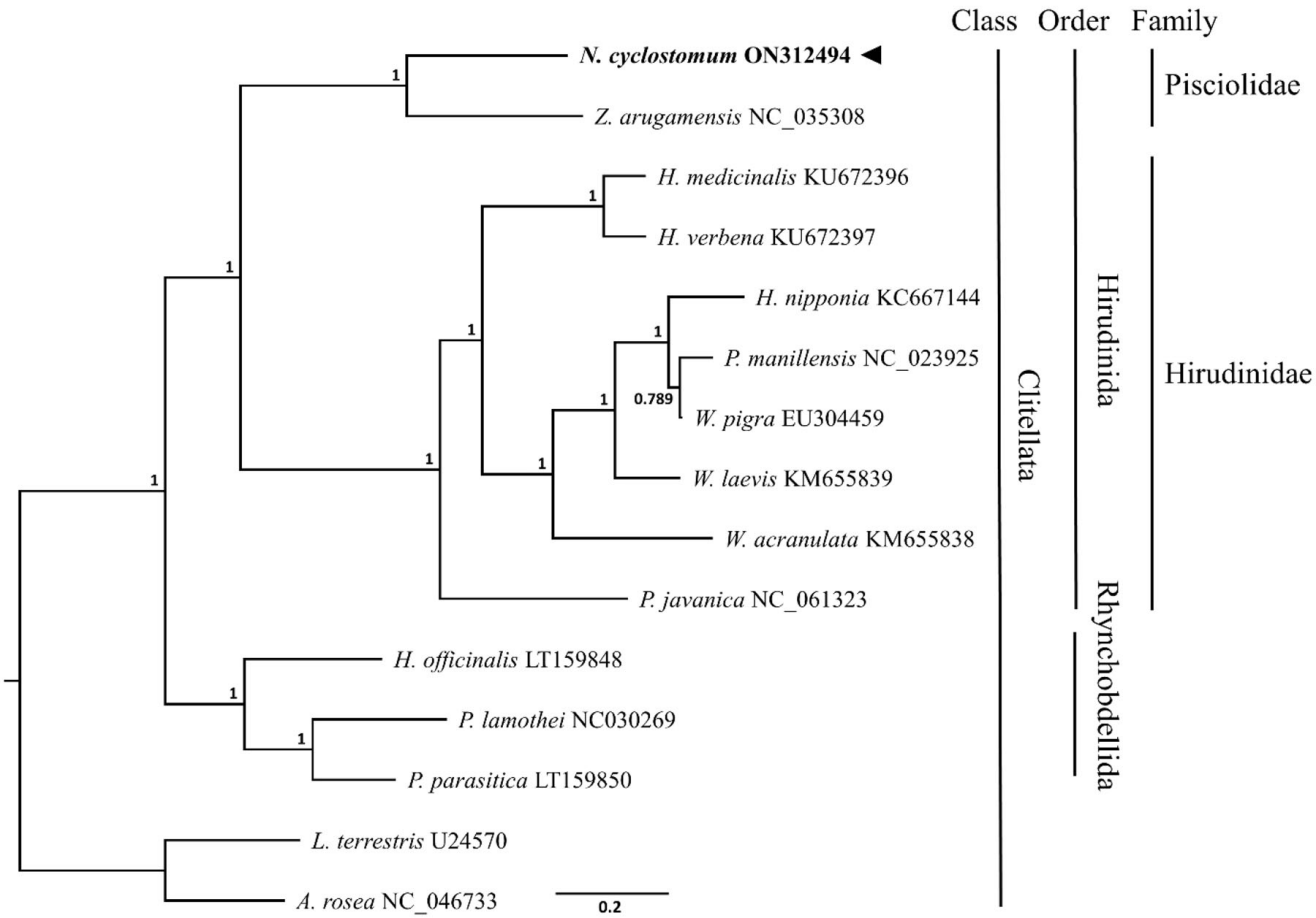
Phylogenetic tree of *Notostomum cyclostomum* and related species. Phylogenetic tree based on the nucleotide sequence of 13 mitochondrial PCGs using Bayesian inference (BI) was conducted on 15 available mitogenomic sequences from the class Clitellata including *Lumbricus terrestris* (U24570) and *Aporrectodea rosea* (NC_047733) as outgroups. GenBank accession numbers of mitogenome sequences are given adjacent to the species names. Node numbers indicate posterior probabilities from the Bayesian inference. The black arrow indicates the *N. cyclostomum* analyzed in this study.

## Data Availability

The genome sequence data that supported the findings of this study are openly available in GenBank of NCBI at (https://www.ncbi.nlm.nih.gov/) under the accession no. ON312494. The associated BioProject, SRA, and Bio-Sample numbers are PRJNA836134, SRR19136323 and SAMN28132021, respectively.
